# The Light and Shadow of Rapid Serological Tests for SARS-CoV-2 Infection: Results from a Study in a Large Emergency Department

**DOI:** 10.3390/ijerph17186493

**Published:** 2020-09-07

**Authors:** Daniela Loconsole, Francesca Centrone, Caterina Morcavallo, Silvia Campanella, Anna Sallustio, Michele Quarto, Vito Procacci, Maria Chironna

**Affiliations:** 1 Department of Biomedical Sciences and Human Oncology-Hygiene Section, University of Bari, Piazza G. Cesare 11, 70124 Bari, Italy; daniela.loconsole@uniba.it (D.L.); francesca.centrone@uniba.it (F.C.); caterina.morcavallo@uniba.it (C.M.); campanella.silvia@libero.it (S.C.); michele.quarto@uniba.it (M.Q.); 2 Hygiene Unit, Policlinico Hospital, Piazza G. Cesare 11, 70124 Bari, Italy; annasallustio@libero.it; 3 Emergency Department, Policlinico Hospital, Piazza G. Cesare 11, 70124 Bari, Italy; v.procacci59@gmail.com

**Keywords:** SARS-CoV-2 infection, COVID-19, serological test, laboratory test, real-time PCR

## Abstract

A critical point in the management of the SARS-CoV-2 pandemic is the need to promptly identify the greatest number of infected people and to implement strict public health measures. In this study, the performance of a rapid serological test in a clinical setting was evaluated. Samples from 819 consecutive patients (with or without respiratory symptoms) admitted to a large Emergency Department were tested between 23 March and 21 April 2020. Patient samples were tested in a real-time PCR assay and a serological assay. In total, 148/819 patients (18.1%) tested positive for SARS-CoV-2 by real-time PCR. The serological test revealed that 70/819 patients (8.5%) had anti-SARS-CoV-2 IgM and/or IgG. The prevalence of anti-SARS-CoV-2 antibodies was significantly higher in patients with respiratory symptoms lasting for >7 days than in those with respiratory symptoms lasting for 0–7 days (*p* < 0.001). The serological assay had an overall sensitivity of 35.1% and an overall specificity of 97.3%. A high negative predictive value (96.7%) was reported for patients without respiratory symptoms. The results confirm that rapid serological assays alone are not sufficient for diagnosis of SARS-CoV-2 infection but can be incorporated into large-scale screening programs during periods in which the virus circulation is low.

## 1. Introduction

In December 2019, the authorities in Wuhan City, China, reported a cluster of pneumonia cases of unknown etiology [1]. On 7 January 2020, a new coronavirus (now known as SARS-CoV-2) was identified as the causative agent. The disease, called COVID-19, spread quickly from Wuhan to other Chinese provinces, and then worldwide. The World Health Organization (WHO) declared COVID-19 a pandemic on 11 March 2020 [2]. As of 6 June 2020, the number of global confirmed diagnoses of SARS-CoV-2 was 6,750,000, with roughly 395,000 deaths [3].

The infection is airborne and transmitted through direct human-to-human contact (exhaled droplets/aerosols) or indirectly through contaminated surfaces [4]. Recently, fecal-oral transmission was hypothesized [5]. SARS-CoV-2 can cause mild to moderate respiratory illness, mostly characterized by fever, cough, and fatigue [6]. Severe cases of pneumonia can progress rapidly to acute respiratory distress syndrome [7]. The incubation period of COVID-19 seems to range from three to seven days, but some cases can be asymptomatic [8]. People infected with SARS-CoV-2 can transmit the virus before they themselves develop significant symptoms [9]. Although the role of asymptomatic carriers is not yet clear, pre-symptomatic/asymptomatic transmission is likely [9,10,11,12].

To date, case isolation, contact tracing, physical distancing, and strict hygiene measures are the only approaches available to slow/stop the pandemic [13]. Thus, a timely and accurate diagnosis based on clinical history and laboratory test data is crucial to contain the virus [4,14,15], to increase our understanding of the virus’ epidemiology, to optimize case management, and to minimize the risk of large-scale outbreaks, particularly in hospitals, nursing homes and prisons [11,14,16]. Currently, identification of SARS-CoV-2 RNA by PCR-based methods is the gold standard method for detecting the virus [17]. However, this process takes several hours and harbors a not insignificant risk of false-negative results depending on the sample type (nasal or pharyngeal swab, sputum), skill in sample collection, or stage of infection (according to the SARS-CoV-2 shedding pattern); however, the actual false-negative rate has not yet been established [18,19,20,21]. Several recent studies indicate that clinical evaluation, traditional imaging, and computed tomography should also be used as complementary approaches to a laboratory diagnosis in order to avoid delay in the management of patients suspected of having SARS-CoV-2 infection [22,23].

Compared with molecular tests, serological assays are easier to perform and have a faster turn-around time [15]; however, the clinical value of antibody responses to SARS-CoV-2 infections is unknown [15]. Recently, several companies have developed rapid qualitative or semi-quantitative in vitro diagnostics (IVDs) [24] to detect IgM and IgG anti-SARS-CoV-2 antibodies in blood samples. These tests are quick (15–30 min), low-cost [25], simple to perform and interpret, and would allow mass screening to facilitate containment of the epidemic; however, they are likely to suffer from poor sensitivity [26]. Therefore, based on currently available data, the WHO recommends use of rapid antibody detection tests only for research purposes [27].

The aim of the present study was to investigate the performance of a rapid colloidal gold-based immunochromatographic test for anti-SARS-CoV-2 antibodies in a large cohort of patients (with or without symptoms of COVID-19) admitted to an Emergency Department (ED) in Southern Italy to assess the reliability of such a test in a clinical setting.

## 2. Materials and Methods 

### 2.1. Study Population

The study population comprised a convenience sample of consecutive series of patients (*n* = 819) who were admitted to the ED of the Policlinico Hospital of Bari, Italy, between 23 March and 21 April 2020. To deal with the COVID-19 pandemic, the ED of Policlinico Hospital (average annual accesses: 110,000) established a “grey zone” for all the patients attending the ED until the results of SARS-CoV-2 molecular test. According to the results, they were transferred to a “COVID” or a “NO-COVID” ward. For analysis purposes, based on the case definition described in the documents of the Italian Ministry of Health (http://www.trovanorme.salute.gov.it/norme/renderNormsanPdf?anno=2020&codLeg=73669&parte=1%20&serie=null), patients were divided into two groups: those admitted to the ED with symptoms suggestive of COVID-19, mainly respiratory symptoms (termed the “RS group”), and those admitted to the ED with acute clinical conditions but no respiratory symptoms (termed the “non-RS group”). The RS group was divided into two sub-groups according to the time between symptom onset and sample collection: the 0–7 days-RS group and the >7 days-RS group. In addition, when available, clinical information (e.g., presence of any underlying chronic disease and the day of symptom onset) was collected. At the time of admission, all patients attending the ED of the Policlinico Hospital were subjected both to a blood draw for serological investigation and to respiratory sample collection (nasal and pharyngeal swabs) for real-time PCR assay of SARS-CoV-2. All samples were analyzed at the Laboratory of Molecular Epidemiology and Public Health of the Hygiene Unit of the Policlinico Hospital Bari, which is the Regional Reference Laboratory for surveillance and diagnosis of SARS-CoV-2. 

### 2.2. Rapid Test for SARS-CoV-2 IgM/IgG

The serologic assay was a rapid colloidal gold-based immunochromatographic test (VivaDiagTM, Vivacheck Biotech, Hangzhou, China). Briefly, 10 µL of plasma or whole blood was applied to the sample well, followed by two drops of dilution buffer. The results were read after about 15 min. If only the quality control line “C” was colored, then the test was interpreted as negative. If the quality control line “C” and the detection IgM and/or IgG lines were colored, then the test was interpreted as positive for IgM and/or IgG anti-SARS-CoV-2 antibodies. If the quality control line “C” was not colored, the test was interpreted as invalid and repeated. The sensitivity declared by the test company was 81.3% after 4–10 days from symptom onset and 97.1% after 11–24 days.

### 2.3. Molecular Identification of SARS-CoV-2

Nasal and pharyngeal swabs (FLOQSwabs^TM^, Copan Italia, Brescia, Italy) were collected from all enrolled patients. RNA was extracted using the Microlab Nimbus automated extraction system (Seegene, Seoul, Republic of Korea), according to the manufacturer’s instructions. A commercial multiplex real-time PCR kit (Allplex™ 2019-nCoV Assay, Seegene, Seoul, Korea) was then used to detect the E, RdRP, and N genes of SARS-CoV-2. Results were considered positive when two or three genes were identified. The WHO Real-time RT-PCR protocol (https://www.who.int/docs/default-source/coronaviruse/uscdcrt-pcr-panel-for-detection-instructions.pdf?sfvrsn=3aa07934_2) was used to confirm results when samples resulted positive for one gene.

### 2.4. Data Analysis

Data analysis was performed using demographic and clinical information collected during the visit. To establish the specificity, sensitivity, positive predictive value (PPV), and negative predictive value (NPV) of the rapid test for SARS-CoV-2 IgM/IgG, the results were compared with those from the real-time PCR. The Kappa coefficient was calculated to evaluate the concordance between the two tests [28]. Data analysis was performed using STATA 12.0 software (StataCorp LLC, Georgetown, TX, USA). The Chi-squared test was used to compare proportion and 95% confidence intervals were reported. A *p*-value of <0.05 was considered significant. 

### 2.5. Ethical Statement

All procedures performed in the study were in accordance with the ethical standards of the institutional and national research committee and with the 1964 Helsinki declaration and its later amendments or comparable ethical standards. Ethical approval was obtained from the Apulian Regional Observatory for Epidemiology (Prot. AOO_005/PROT/0001546). Informed written consent was obtained from all individuals or legal guardian of patients who provided the specimens.

## 3. Results

Of the 819 patients enrolled in the study, 454 (55.4%) were male. The median age was 66 years (IQR: 52–80). Among these patients, no case of a pre-existing SARS-CoV infection or anti-SARS vaccination was reported. The characteristics and the distribution of patients by age group is shown in Table 1.

Of the whole, 721 patients (88.0%) were classified into the RS group and 98 (12.0%) into the non-RS group. The demographic characteristics of the two groups were comparable (Table 2). The date of respiratory symptom onset was known for 610/721 patients (84.6%). The average time between illness onset and sample collection was 3.9 days (range, 0–60 days).

In the overall population, 148/819 patients (18.1%) tested positive for SARS-CoV-2 by real-time PCR and 70/819 (8.5%) had IgM and/or IgG antibodies against SARS-CoV-2. The overall results of serological and molecular tests for SARS-CoV-2 for all enrolled patients are shown in Table S1.

Among the 721 patients belonging to the RS group, 143 (19.8%) were positive for SARS-CoV-2 by molecular test (Table 3). SARS-CoV-2 was detected in 5/98 patients (5.1%) in the non-RS group. Among patients with a positive PCR result for SARS-CoV-2, 24/99 (24.2%) of those in the 0–7 days-RS group and 26/44 (59.1%) in the >7 days-RS group had IgM and/or IgG anti-SARS-CoV-2 antibodies (*p* < 0.001). Five patients with no respiratory symptoms had a positive PCR test result for SARS-CoV-2, and two of these had anti-SARS-CoV-2 antibodies. Among patients resulting positive by molecular test (n = 148) in particular, 52 (35.1%) were positive also for IgM and/or IgG. Of samples positive by rapid serological test, where 52/70 (74.3%) were also positive by real-time PCR. 

IgM and/or IgG anti-SARS-CoV-2 were detected in 70/819 patients (8.6%). In particular, IgM and IgG were both detected in 59/70 patients (84.3%), only IgM in 10/70 patients (14.3%), and one case was positive only for IgG. Among patients resulting positive by rapid serological assay, 32/70 (45.7%) were symptomatic from >7 days, 28/70 (40.0%) were symptomatic from 0–7 days, and 6/70 (8.6%) were asymptomatic. The data are not available for four patients.

According to the reported data, the SARS-CoV-2 VivaDiag^TM^ serological assay had an overall sensitivity of 35.1% and an overall specificity of 97.3%. For the whole population, the reported PPV of the serological rapid test was 74.3% and the reported NPV was 87.2%. The Kappa coefficient [28] was 0.41, showing a moderate concordance between the two tests. Sensitivity, specificity, PPV, and NPV in the RS and non-RS groups are shown in Table 4. In particular, the rapid serological assay for anti-SARS-CoV-2 antibodies showed the highest sensitivity in the >7 days-RS group (57.1%), the highest specificity in the 0–7 days-RS group (99.3%), the highest PPV in the 0–7 days-RS group (88.9%), and the highest NPV in the non-RS group (96.7%).

## 4. Discussion

The ongoing COVID-19 pandemic has revealed the importance of rapid and reliable laboratory diagnosis of SARS-CoV-2 infection to limit its spread; however, our knowledge about diagnostic tests is still evolving [29]. Molecular tests are still considered the gold standard for confirming SARS-CoV-2 infection [17], but the role of serological tests should be considered for patients presenting late at diagnosis to understand the extent of COVID-19 spread in the community, and to identify subjects exposed and naturally immune to the virus [29]. Preliminary evidence suggests that infected individuals generate antibodies; some studies suggest within five days [25], whereas others suggest from the second week after symptom onset [27]. The results of our study showed that the percentage of patients positive for IgM and/or IgG with symptom onset from >7 days was almost the same as of patients positive for IgM and/or IgG with symptom onset from 0–7 days (45% vs. 40%). Based on this result, the date of symptom onset could not be used to hypothesize the timing of seroconversion since several days may elapse between the date of infection and occurrence of clinical signs. 

Even though high titers of IgG antibodies seem to correlate positively with the presence of neutralizing antibodies [30], it is not clear whether these antibodies are protective or for how long the protection lasts [31]. Here, we found that only 8% of the whole population had IgM and/or IgG antibodies against SARS-CoV-2. However, this low percentage could be related to the short period between onset of symptoms and the date of sampling. In fact, the percentage of patients in the >7 days-RS group who had antibodies increased to nearly 60%. Some studies report that some subjects with SARS-CoV-2 infection confirmed by molecular tests have a delayed or even absent antibody response [15,32]. Moreover, the strength of an antibody response seems to be related to factors such as age, nutritional status, severity of clinical manifestations, the presence of other infections (e.g., HIV infection), or therapies that suppress the immune system [15,27]. More than half the population in the present study were aged >65 years, and more than 60% of these had at least one underlying chronic condition. It should be borne in mind that these conditions could have interfered with the immune response. 

A relevant finding of this report is that six subjects belonging to the non-RS group tested positive in the rapid serological test and two of these were confirmed as positive for SARS-CoV-2 RNA. Moreover, 5% of asymptomatic patients were positive at PCR test. This is relevant in light of the role of asymptomatic or pre-symptomatic patients in spreading the virus because these subjects play a major role in transmission [11]. 

The rapid SARS-CoV-2 serological test has several advantages. First, it is quick, cheap, and simple to perform at the bedside and does not require specialist equipment [33]. However, use of serological assays in the absence of molecular tests would result in the majority of patients that tested positive by real-time PCR being identified as negative. The results of the present study show that the overall sensitivity and specificity of the serological test were 35.1% and 97.3%, respectively. Our results are in agreement with other Italian studies of the clinical benefits of rapid serological assays in hospital settings [34,35]. Moreover, a meta-analysis by Bastos et al. states that evidence does not support the use of the serological test at the point-of-care of hospital setting because of the high risk of bias and limited generalizability [36]. Our study shows that, compared with people showing symptoms from 0–7 days, subjects showing symptoms after more than seven days are significantly more likely to have detectable antibodies. Nevertheless, the rapid test for SARS-CoV-2 IgM/IgG shows suboptimal sensitivity (<60%) also in people showing symptoms for more than seven days, confirming that serological tests alone are not sufficient for diagnosis nor for triage of patients with suspected COVID-19, as previously reported [27,34]. However, we must not discount the occurrence of false-negative results from the molecular test [7], in particular in the >7 days-RS group, because of the longer time elapsed between symptom onset and the sample collection. This issue could affect specificity in this group. To significantly improve the sensitivity of the diagnosis, combined detection of RNA and anti-SARS-CoV-2 antibodies should be considered in clinical practice [15]. 

The present study was conducted during a period characterized by a high prevalence of COVID-19 in Italy [3]. The performance of the screening tests is better when the prevalence of the disease in the population is high. In fact, the rapid IgM/IgG test shows a high PPV in subjects with symptoms suggestive of COVID-19 and an NPV of almost 97% in subjects without respiratory symptoms. However, as suggested by Li et al., the serological rapid test for SARS-CoV-2 could be an effective screening tool for use by businesses, schools, airports, seaports, and train stations; thus, such tests have the potential to be a powerful weapon against this global threat [33]. These data suggest the possible use of these kinds of serological assays in settings other than hospitals; for example, for large-scale screening of asymptomatic subjects during a period of low SARS-CoV-2 circulation. In contrast to this position, other studies report that the very low sensitivity of these tests means that they should neither be used for community screening nor for public health measures [26]. Based on the high NPV reported in our study, we could speculate that the rapid serological assay should be considered for a large-scale screening program. 

This study has some limitations. First, there is no comparison with an immunoenzymatic assay for identification of specific antibodies against SARS-CoV-2. Moreover, the possible cross-reactivity of the rapid test with other coronaviruses or influenza viruses was not considered [34]. However, the strength of the present study is that, compared with other studies, the study population is larger. Therefore, we can speculate that our results may be considered more reliable.

## 5. Conclusions

The results of our study confirm that, due to low sensitivity, rapid serological assays should not be considered as an alternative to molecular tests as a diagnostic tool in hospital settings; rather, they could be used together with real-time PCR to improve the sensitivity of the diagnosis and to complete the diagnostic algorithm (as happens for other infectious diseases). However, because of the high NPV in the asymptomatic population, this test could be considered for screening of the general population, especially in a period of low SARS-CoV-2 circulation, to identify asymptomatic carriers of SARS-CoV-2 or to evaluate the extent of previous SARS-CoV-2 infections. Further high-quality clinical studies are needed to assess the reliability of this tool in a large-scale screening also.

## Figures and Tables

**Table 1:** Characteristics of the 819 enrolled patients in the Emergency Department (23 March–21 April).

Characteristic	*N*	% (95% CI)
Gender		
Male	454	55.4% (0.520–0.588)
Female	365	44.6% (0.412–0.479)
Age group (years)		
≤35	78	9.5% (0.077–0.117)
36–55	170	20.8% (0.181–0.237)
56–65	150	18.3% (0.158–0.212)
>65	421	51.4% (0.479–0.548)
Chronic disease		
Yes	516	63.0% (0.596–0.663)
No	150	18.3% (0.158–0.212)
Unknown	153	18.7% (0.161–0.216)

*N*, number; CI, confidence interval.

**Table 2:** Comparison of demographic and clinical characteristics of patients admitted to the Emergency Department with (RS group) and without (Non-RS group) respiratory symptoms.

Characteristics	RS Group		Non-RS Group		*p*-Value
	*N*	% (95% CI)	*N*	% (95% CI)	
Total	721	88.0% (0.856–0.900)	98	12.0% (0.099–0.144)	
Gender					
Male	401	55.6% (0.519–0.592)	53	54.1% (0.443–0.636)	*p* = 0.77
Female	320	44.4% (0.408–0.480)	45	45.9% (0.364–0.558)	
Chronic disease					
Yes	446	61.9% (0.582–0.653)	67	68.4% (0.586–0.767)	*p* = 0.86
No	127	17.6% (0.15–0.206)	20	20.4% (0.136–0.294)	
Unknown	148	20.5% (0.177–0.236)	11	11.2% (0.064–0.189)	
Symptom onset (days)					
0–7	514	71.3% (0.679–0.745)	-	-	
>7	96	13.3% (0.110–0.159)	-	-	
Unknown	111	15.4% (0.129–0.183)	-	-	

*N*, number; RS, respiratory symptoms; CI, confidence interval.

**Table 3:** Results of serological and molecular tests for SARS-CoV-2 of all patients enrolled.

	RS Group		Non-RS Group		*p*-Value
	*N*	% (95% CI)	*N*	% (95% CI)	
Real-time PCR					
Positive	143	19.8% (0.171–0.229)	5	5.1% (0.022–0.114)	*p* = 0.0003
Negative	578	80.2% (0.771–0.829)	93	94.9% (0.886–0.978)	
Rapid serological test					
Positive	64	8.9% (0.07–0.112)	6	6.1% (0.028–0.127)	*p* = 0.36
Negative	657	91.1% (0.888–0.929)	92	93.9% (0.873–0.972)	

RS, respiratory symptoms; PCR, polymerase chain reaction; CI, confidence interval.

**Table 4:** Diagnostic performance of the rapid serological test versus the real-time PCR reference standard in the RS and Non-RS groups.

	Sensitivity (95% CI)	Specificity (95% CI)	PPV (95% CI)	NPV (95% CI)
RS group	34.9% (0.299–0.387)	97.8% (0.965–0.987)	79.4% (0.679–0.879)	85.9% (0.848–0.867)
0–7 days	24.5% (0.194–0.267)	99.3% (0.981–0.998)	88.9% (0.705–0.971)	84.8% (0.838–0.853)
>7 days	57.1% (0.455–0.661)	85.2% (0.761–0.921)	75.0% (0.597–0.867)	71.9% (0.642–0.777)
Non-RS group	40.0% (0.076–0.797)	95.7% (0.94–0.978)	33.3% (0.063–0.664)	96.7% (0.95–0.989)

CI, confidence interval; RS, respiratory symptoms; PPV, positive predictive value; NPV, negative predictive value.

## Supplementary Materials

The following are available online at https://www.mdpi.com/1660-4601/17/18/6493/s1. Table S1: Results of serological and molecular tests for SARS-CoV-2 for all enrolled patients.
