# Supplementary material for: The Light and Shadow of Rapid Serological Tests for SARS-CoV-2 Infection: Results from a Study in a Large Emergency Department
Source: Int J Environ Res Public Health. 2020 Sep 7;17(18):6493. doi: 10.3390/ijerph17186493 (PMC7558531; doi:10.3390/ijerph17186493)
Supplement: Supplementary file 1 [file ijerph-17-06493-s001.pdf]

Supplementary material.

Table S1. Results of serological and molecular tests for SARS-CoV-2 for all enrolled patients.

No. of patients	Real-time PCR	IgM	IgG
n = 148	**Positive**		
	n = 47	+	+
	n = 4	+	-
	n = 1	-	+
	n = 96	-	-
n = 671	**Negative**		
	n = 653	-	-
	n = 12	+	+
	n = 6	+	-
	n = 0	-	+

PCR, polymerase chain reaction.
